# News and Coming Events

**Published:** 1909-04-17

**Authors:** 


### News and Coming Events.

Alderman Leon Emanuel, of Portsmouth, twice mayor of that borough, left £10,500 for charitable purposes, of which £1,000 was bequeathed to the Portsmouth Eye and Ear Infirmary.

It is announced that the Army Council has approved the appointment of Dr. W. S. A. Griffith, M.D., F.R.C.P., F.R.C.S., of St. Bartholomew's Hospital, to be consulting gynæcologist to the Queen Alexandra's Military Hospital.

The Lord Mayor, accompanied by the Sheriffs, will visit St. Bartholomew's Hospital on Friday, May 7, at 3 p.m. to open the new Pathological Block. The ceremony will take place in the Great Hall of the Hospital.

We regret to learn of the death, which occurred this week at 10 Stratford Place, after a long illness, of Louisa, wife of Sir Lauder Brunton, Bart., M.D., and daughter of the late Ven. T. A. Stopford, Archdeacon of Meath.

Professor G. Sims Woodhead, M.D., F.R.S. Ed., etc., has been appointed to serve upon the Committee appointed by the Treasury to consider the claims to additional State assistance and estimates supplied by the Scottish Universities at the request of his Majesty's Government; and to report as to what assistance, if any, should be granted from public funds in the interests of the proper development of the work of the Universities.

Dr. F. J. Waldo recently at a Southwark inquest objected to the word "temporarily" in a verdict of suicide while insane. He said he could not record the word, and the jury eliminated it. The Coroner said he knew the word was used, but he considered it wrongly used. A Judge in the Appeal Court had declared that "temporary insanity" was unknown to English law. Dr. Waldo said that the medical term was "impulsive insanity."

The new out-patient department and nurses' home of the Royal National Orthopædic Hospital is to be opened on Tuesday, April 20, at 3.45, by Princess Alexander of Teck, and the arrangements for the ceremony are now almost complete. Tea is to be served on the ground floor in the gymnasium and massage rooms and the formal opening ceremony will take place in the out-patient waiting hall. Mr. Alfred de Rothschild has kindly consented to allow Mr. Carl Heubert's Viennese Orchestra to play on the occasion. Her Royal Highness will be received by the Duke of Marlborough, Lord and Lady Denbigh, Sir Richard and Lady Martin, and other members of the committee and ladies' committee of the hospital. Admission will be by ticket, applications for which should be addressed to the secretary, 254 Great Portland Street, W.

Four lectures will be delivered at Gresham College, Basinghall Street, E.C., on Tuesday, April 20; Wednesday, April 21; Thursday, April 22; and Friday, April 23, 1909; by Dr. F. M. Sandwith, M.D., F.R.C.P., Gresham Professor of Physic. The lectures are free to the public, and will begin each evening at six o'clock. Lecture I. will deal with "The History of Cancer"; Lecture II. with "The Geographical Distribution and Theories of Causation of Cancer" (illustrated by lantern slides); Lecture III. with "The Causes, Symptoms, and Treatment of Cancer"; and Lecture IV. with "The Results of Recent Research on Malaria, Yellow Fever, Trypanosome Infections, Malta Fever, and Diphtheria" (this lecture also will be illustrated by lantern slides).

Inspector-General James Porter, C.B., M.D., Director-General of the Medical Department of the Navy, has been appointed Honorary Physician to the King, in the place of Inspector-General Sir John Watt Reid, K.C.B., deceased.

The delegates of the Common University Fund of Oxford University have appointed Dr. Joseph F. Payne, honorary Fellow of Magdalen College, to deliver six lectures next term on "The History of Greek Medicine up to the Age of Hippocrates," at the schools, on Wednesdays and Fridays, at 5.45 p.m., beginning on May 5.

Mr. H. A. T. Fairbank, M.S., F.R.C.S., surgeon to out-patients at Great Ormond Street Hospital and orthopœdic surgeon and demonstrator of anatomy at Charing Cross Hospital, has been appointed surgeon to the Miller General Hospital for South East London, Greenwich Road, S.E., in succession to Mr. John Mackern, M.D., F.R.C.S., who has recently resigned.

### ROYAL COLLEGE OF PHYSICIANS OF LONDON.

At an extraordinary comitia on April 5, Sir R. Douglas Powell, Bart., K.C.V.O., President, in the chair, Dr. W. Pasteur was appointed representative of the College at the 350th anniversary of the foundation of the University of Geneva, to be held in July next. The President, after thanking the College for its expression of sympathy on the death of Lady Powell, delivered his Annual Address, in which he offered congratulations to those of the College who had received Royal honours during the past year, and referred to the awards of the College and to the oration and lectures which had been delivered. He mentioned the gifts to the College and the increased number of colonial graduates admitted to the examinations, and also to the action which had been taken to oppose the Charter (or certain of its clauses) which was being asked for by the British Medical Association. The President then read obituary notices of those Fellows who had died during the past year, and concluded by thanking the Officers, Censors, and Fellows of the College for their services during his year of office. A vote of thanks to Sir R. Douglas Powell for his services during the past year was carried with acclamation; and at the election for President he was re-elected by a very large majority. After some discussion and the consideration of various minor matters, a by-law for the admission of women to the examinations of the College for qualifications was enacted for the first time as follows: "Women shall be eligible for admission as licentiates and members of the College and for the grant of a diploma in public health on the same terms and conditions as men, and so far as is necessary to give effect to this by-law, words in the by-laws and regulations importing the masculine gender shall include females, and all proper alterations shall be made in the forms of the Letters Testimonial and the Licence granted by the College. *Provided always* that women shall not be eligible for election as Fellows of the College or be entitled to take any part in the government, management, or proceedings of the College." The proposed commemoration of the 400th anniversary of the birth of Dr. Caius, formerly President and benefactor of the College, and second founder of Gonville and Caius College, Cambridge, was discussed; and finally a report from the Committee of Management recommending that the University of St. Andrews be added to the institutions at which the complete curriculum of professional study for the diplomas of the Royal Colleges may be pursued and whose graduates may be admitted to the final examination of the examining board in England, on production of the required certificates of study, was adopted.

